# The effect of pedicle subtraction osteotomy for the correction of severe Scheuermann thoracolumbar kyphosis on sagittal spinopelvic alignment

**DOI:** 10.1186/s12891-020-03942-7

**Published:** 2021-02-10

**Authors:** Guanfeng Lin, Shengru Wang, Yang Yang, Zhe Su, You Du, Xiaolin Xu, Xiran Chai, Yipeng Wang, Bin Yu, Jianguo Zhang

**Affiliations:** Departments of Orthopaedic Surgery, Peking Union Medical College Hospital (PUMCH), Peking Union Medical College and Chinese Academy of Medical Sciences; State Key Laboratory of Complex Severe and Rare Diseases, Peking Union Medical College Hospital(PUMCH), Peking Union Medical College and Chinese Academy of Medical Sciences, No. 1 Shuaifuyuan Hutong, Beijing, 100730 People’s Republic of China

**Keywords:** Scheuermann kyphosis, Severe thoracolumbar kyphosis, Pedicle subtraction osteotomy, Sagittal balance, Spinopelvic parameters

## Abstract

**Purpose:**

To analyze how pedicle subtraction osteotomy (PSO) treatment of severe Scheuermann thoracolumbar kyphosis (STLK) using pedicle screw instrumentation affects sagittal spinopelvic parameters.

**Background:**

The medical literature on the post-surgical effects of treatments such as Ponte osteotomy is limited, but suggests few effects on spinopelvic profiles. Currently, there is no research regarding changes in sagittal spinopelvic alignment upon PSO treatment in STLK patients.

**Methods:**

We performed a retrospective study on 11 patients with severe STLK. These patients underwent posterior-only correction surgeries with PSO and pedicle screw instrumentation between 2012 to 2017 in a single institute. Patients were measured for the following spinopelvic parameters: global kyphosis (GK), thoracic kyphosis (TK), thoracolumbar kyphosis (TL), lumbar lordosis (LL), sagittal vertical axis (SVA), pelvic incidence (PI), pelvic tile (PT), sacral slope (SS), and administered a Scoliosis Research Society-22 questionnaire (SRS-22) pre-operation, post-operation and at final follow-up.

**Results:**

GK improved from a median of 74.1° to 40.0° after surgery, achieving a correction rate of 48.8% with a median correction loss of 0.8°. TK, TL and LL all showed significant difference (*P* < 0.05) and SVA improved 22.7 (11.6, 30.9) mm post operation. No significant difference was found in pelvic parameters (PI, PT, SS, all *P* < 0.05). The absolute value of LL- PI significantly improved from a median of 26.5° pre-operation to 6.1° at the final follow-up. 72.7% in this series showed an evident trend of thoracic and lumbar apices migrating closer to ideal physiological segments after surgery. Self-reported scores of pain, self-image, and mental health from SRS-22 revealed significant improvement at final follow-up (all *P* < 0.05).

**Conclusions:**

PSO treatment of severe STLK with pedicle screw instrumentation can improve spine alignment and help obtain a proper alignment of the spine and the pelvis.

Scheuermann kyphosis (**SK**) is a structural hyperkyphotic deformity that accounts for the most common cause of sagittal spinal deformities in adolescents. The presence of at least three consecutive vertebral bodies with anterior wedging more than 5° is conventionally used to confirm a diagnosis of SK [1]. SK can be classified into two types: typical and atypical [2]. The thoracic type (or typical) accounts for the majority of cases and its apex usually located in the mid-thoracic spine. In contrast, the apex of the thoracolumbar type (or atypical, abbreviated as **STLK**) falls in the thoracolumbar spine. Most current studies do not distinguish between the two forms of kyphosis in their investigations.

In recent years, the importance of sagittal spinopelvic alignment has acquired extensive interest among spine surgeons, as it is widely accepted that the maintainence and restoration of sagittal spinopelvic balance is critical to quality of life after spinal surgeries [3–6]. SK often presents with thoracic or thoracolumbar hyperkyphosis, compensatory cervical and lumbar hyperlordosis, and altered sagittal plane alignment [7]. STLK is more likely to progress and become symptomatic during adulthood [8]. The lower location of kyphosis in STLK tends to have a greater influence on the pelvic morphology compared to typical SK. In the two different types of SK, patients adopt distinct compensatory mechanisms to keep the sagittal balance, and different treatment strategies should be taken in consideration depending on type classification [9]. Analysis of spinopelvic parameters and SK has mainly been performed in nonsurgical patients, with much more limited research taking surgeries and sagittal spinopelvic alignment into consideration. Surgical treatment for SK by Ponte osteotomy appear to have little or no influence on changing spinopelvic profiles [10].

To our knowledge, there is so far no literature concerning changes in sagittal spinopelvic alignment when pedicle subtraction osteotomy (**PSO**) is performed in severe STLK. Furthermore, few studies have focused on the alignment of spine and pelvis. This study aims to evaluate how PSO treatment of severe STLK influences sagittal balance and the alignment of spine and pelvis.

## Materials and methods

With the approval of the institutional review board of the hospital, we identified patients with SK at a single institution from January 2012 to December 2017. Patients participating in our study met the following criteria: (1) Present with radiographic findings such as Schmorl nodes, a result of flattening of the vertebral end plates and narrowing of the intervertebral disc spaces, with or without consecutive wedging vertebra; (2) apex of kyphosis located in thoracolumbar area; (3) undergone posterior-only procedure with PSO and pedicle-screw instrumentation; (4) follow-up >2 years. Exclusion criteria included: (1) any other spinal abnormality in addition to SK, (2) any previous spinal trauma or surgery, (3) incomplete follow-up materials and poor radiographic images.

Indications for surgery included progressive kyphosis or pain, failure of conservative treatment, and inability to accept physical appearance with psychological distress.

### Radiographic measurements

Radiographic measurements were conducted independently by two separate researchers and the mean values were combined for analyses. Radiographic parameters were measured on the preoperative, immediate postoperative, and final follow-up radiographs. For each patient, the following parameters were measured and recorded:

① Global kyphosis (**GK**), the maximum sagittal kyphosis Cobb angle
② Thoracic kyphosis (**TK**), the Cobb angle between T2 and T12
③ Thoracolumbar kyphosis (**TL**), the Cobb angle between T10 and L2
④ Lumbar lordosis (**LL**), the Cobb angle between L1 and S1
⑤ Sagittal vertical axis (**SVA**), the horizontal distance from the C7 plumb line to the posterior superior corner of S1
⑥ Pelvic incidence (**PI**), the angle between the line perpendicular to the upper sacral plate at its midpoint and the line connecting this point to the middle femoral head axis
⑦ Pelvic tilt (**PT**), the angle between the vertical and the line connecting the midpoint of the sacral plate to the middle femoral head axis
⑧ Sacral slope (**SS**), the angle between the horizontal plane and the sacral endplate

### Surgical technique

The patient was placed prone on a radiolucent spinal frame and the thoracolumbar area was approached posterior after intubation and general anesthesia. After surgical exposure, pedicle screws were placed with the free hand technique at all the levels. Size of the screws was chosen depend on development of vertebras (generally 6.0*35 mm for T5–6, 6.0*40 mm for T7–10, 6.0*45 mm for T11-L4). The trajectories of all the screws were confirmed by a C-arm image intensifier. PSO as previously described [11, 12] was performed at the apex vertebra. Multilevel facetectomies were employed distal to the PSO level if necessary. Prebent and physiologically curve-shaped dual rods were implanted. Correction and restoration were achieved by closure, compression and leverage. Decortication of the posterior elements was performed, followed by bone grafting. Sensory- and motor-evoked potentials were used intraoperatively.

### Patient-reported outcomes

The Scoliosis Research Society-22 questionnaire (**SRS-22**) was used to assess patient outcomes. All questionnaires were completed by patients with the oversight of surgeons preoperatively, postoperatively and at final follow-up.

### Statistical analysis

All analyses were carried out using SPSS version 23.0 (SPSS, Chicago, IL). General statistics were analyzed in terms of medians and interquartile ranges. After the descriptive analysis, Student’s t test was performed in the case of a normal distribution. For data with skewed distributions, the Mann–Whitney-U-test was used. Statistical significance was set at *P* < 0.05.

## Results

In total, there were 51 SK patients receiving surgeries at our institution from January 2012 to December 2017. Thirty-three of them had thoracic type SK, while 18 of them had thoracolumbar type SK. In these 18 STLK patients, 2 received Smith-Peterson osteotomy surgery, 5 received Ponte surgery, while 11 of these severe cases received posterior corrections with PSO and pedicle screw instrumentation. The median age was 16 (14, 24) years old. The follow-up duration was median 35 (26, 57) months. The median operative duration was 250 (245, 270) minutes. The median estimated blood loss was 1200 (950, 1250) mL, and 6 of them had need for perioperative blood transfusions.

Full data of all patients was listed in Table 1. Significant corrections were found at final follow-up (*P* < 0.05) without great correction loss of GK (Table 2). The correction rate of GK immediately post-operation was 49.8% (43.2%, 53.8%). During the follow-up, only a slight correction loss was observed, with a mean correction loss of 0.8° (− 1.7, 4.8).

**Table 1:** Full patient data preoperatively, postoperatively and at the final follow-up

No	Parameters	GK (°)	TK (°)	TL (°)	LL (°)	SVA (mm)	PI (°)	PT (°)	SS (°)
1	Pre	66.2	40.9	59.7	45.9	10.7	41.2	17.7	26.6
	Post	32.0	23.9	3.5	31.5	−10.3	40.8	15.2	25.3
	Follow-up	52.1	42.1	8.1	37.5	20.0	44.9	14.5	29.1
2	Pre	91.5	19.2	91.5	50.5	−15.9	33.1	6.6	28.5
	Post	37.5	23.7	37.5	40.1	30.5	34.2	−2.3	40.5
	Follow-up	38.3	22.8	38.3	37.5	17.0	35.7	−6.3	37.6
3	Pre	70.3	57.0	42.2	56.9	10.0	40.1	20.7	22.6
	Post	33.4	33.4	2.9	37.5	−30.4	41.2	8.1	33.7
	Follow-up	32.5	34.7	1.2	41.3	−20.9	42.9	8.2	30.4
4	Pre	69.5	57.5	37.2	60.9	5.0	40.5	13.9	28.6
	Post	32.1	31.1	1.8	34.9	−10.5	40.0	11.4	24.8
	Follow-up	32.2	34.2	2.9	45.1	−27.6	42.3	14.2	29.5
5	Pre	74.1	61.4	40.1	78.7	−40.5	48.1	13.7	35.4
	Post	44.4	38.3	15.3	56.3	−25.2	50.1	13.0	41.8
	Follow-up	47.1	41.1	16.1	58.2	−28.9	52.8	11.1	37.7
6	Pre	70.8	40.8	62.9	62.8	3.3	36.3	−1.0	37.8
	Post	40.2	31.5	21.1	44.3	−33.6	37.5	5.5	33.9
	Follow-up	45.0	36.0	22.3	39.5	−22.0	40.5	5.2	32.7
7	Pre	85.5	38.5	68.5	56.4	32.6	50.3	20.8	31.5
	Post	36.9	25.0	22.5	40.8	11.3	51.6	15.1	38.1
	Follow-up	35.2	24.7	18.5	55.5	22.8	47.4	12.2	40.2
8	Pre	88.5	76.2	50.0	78.7	7.7	43.5	1.9	42.2
	Post	48.1	41.5	7.6	52.3	−19.3	40.1	5.1	38.9
	Follow-up	45.5	43.1	8.5	55.2	−10.5	41.5	6.7	39.7
9	Pre	79.8	70.5	37.6	87.5	−31.5	36.0	−8.2	44.1
	Post	40.0	39.4	1.9	48.1	13.9	38.1	1.3	30.8
	Follow-up	64.5	61.4	3.5	66.3	−45.0	34.9	−3.9	39.6
10	Pre	66.8	52.0	49.5	57.1	42.8	27.7	−4.3	30.3
	Post	40.1	34.5	15.8	34.0	15.0	29.5	5.7	18.4
	Follow-up	43.2	38.4	18.1	44.5	14.1	25.4	2.3	28.7
11	Pre	84.1	77.4	45.1	72.7	−3.8	39.2	28.3	11.7
	Post	47.1	38.5	8.7	47.5	−27.9	43.3	14.5	28.2
	Follow-up	44.7	43.3	7.9	49.5	−26.5	43.4	13.6	23.1

**Table 2:** Comparison of spinopelvic parameters preoperatively, postoperatively and at the final follow-up

Parameter	Pre-op	Post-op	Follow-up	*P*
GK (°)	74.1 (69.5, 85.5)	40.0 (33.4, 44.4)	44.7 (35.2, 47.1)	< 0.001*
TK (°)	57.0 (40.8, 70.5)	30.4 (25.0, 38.5)	38.4 (34.2, 43.1)	0.002*
TL (°)	49.5 (40.1, 62.9)	8.7 (2.9, 21.1)	8.5 (3.5, 18.5)	< 0.001*
LL (°)	60.9 (56.4, 78.7)	40.8 (34.9, 48.1)	45.1 (39.5, 55.5)	< 0.001*
SVA (mm)	5.0 (−15.9, 10.7)	−10.5 (−27.9, 13.9)	−20.9 (−27.6, 17.0)	0.097
PI	40.1 (36.0, 43.5)	40.1 (37.5, 43.3)	42.3 (35.7, 44.9)	0.136
PT	13.7 (−1.0, 20.7)	8.1 (5.1, 14.5)	8.2 (2.3, 13.6)	0.258
SS	30.3 (26.6, 37.8)	33.7 (25.3, 38.9)	32.7 (29.1, 39.6)	0.165
Absolute value of LL- PI	26.5 (16.8, 33.5)	6.2 (4.5, 10.0)	6.1 (1.8, 13.7)	< 0.001*

All parameters were described by Median (P25, P75). *GK* Global kyphosis, *TK* Thoracic kyphosis, *TL* Thoracolumbar curve, *LL* Lumbar lordosis, *SVA* Sagittal vertical axis, *PI* Pelvic incidence, *PT* Pelvic tile, *SS* Sacral slope, * means significant difference of the parameters between pre-op and final follow-up

All sagittal spinal parameters (TK, TL, LL) except SVA showed significant differences at the final follow-up, compared to pre-operation measurements (all *P* < 0.05) (Table 2). SVA showed no statistical significance between pre-operation and follow-up, but improved 22.7 (11.6, 30.9) mm after surgery.

No significant difference was found in pelvic parameters (PI, PT, SS, all *P* < 0.05, Table 2). There was a large change in the absolute value of LL-PI after surgery, with a final mean of 6.1° (1.8, 13.7) at the follow-up.

**Fig. 1:**
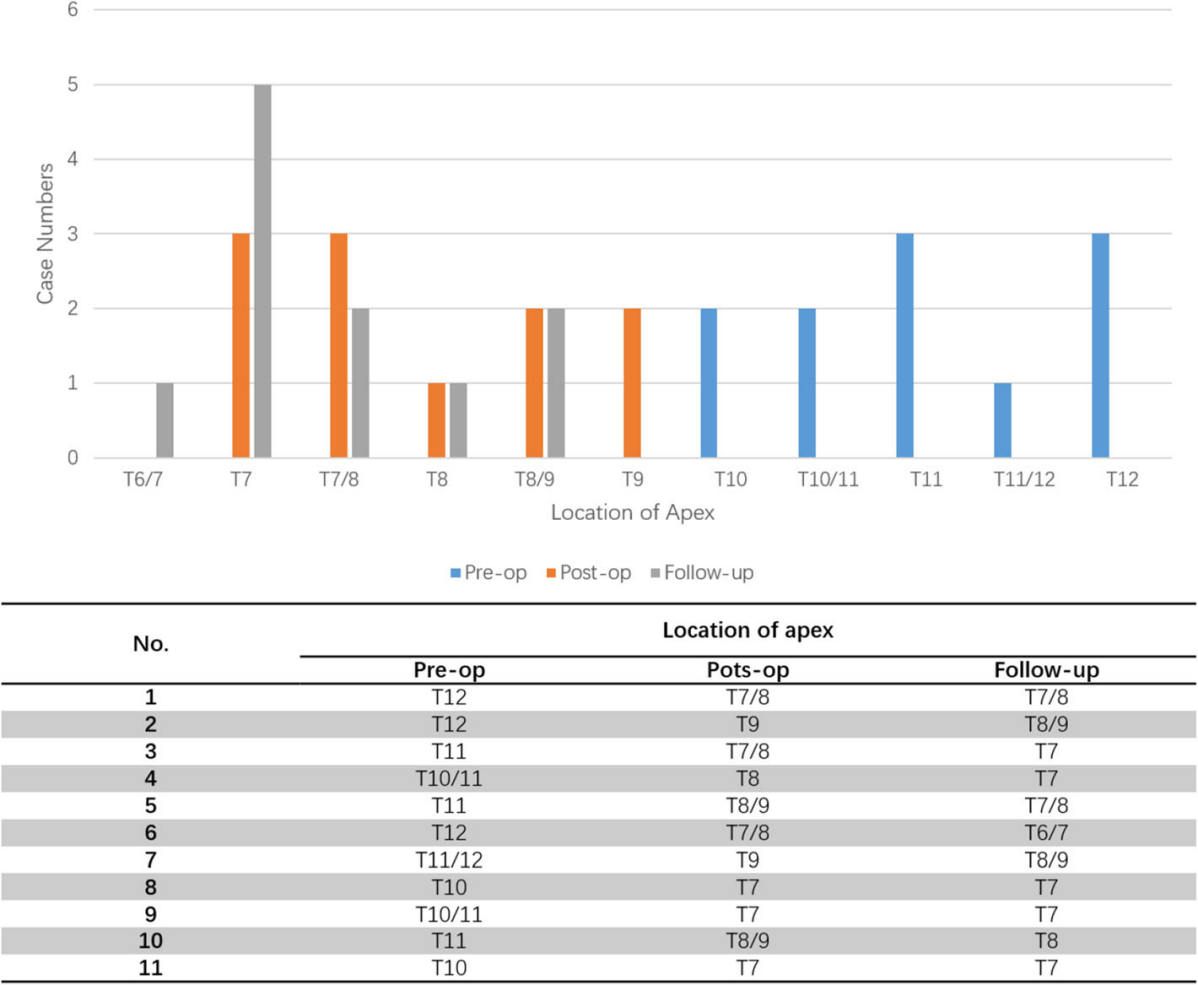
Comparison of the distribution of the thoracic apex preoperatively, postoperatively and at the final follow-up

**Fig. 2:**
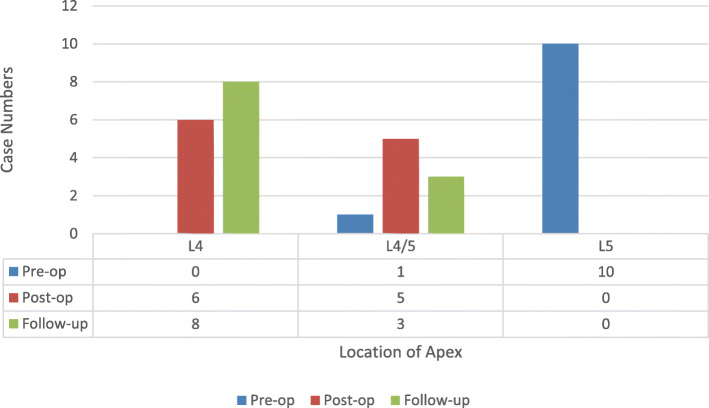
Comparison of the distribution of the lumbar apex preoperatively, postoperatively and at the final follow-up

We observed an evident trend (Figs. 1, 2) of thoracic and lumbar apex migration closer to ideal physiological positions after surgery. While the apex location of sagittal kyphosis was around T10–12 pre-operation, this localization was normalized to T7–8 in 72.7% of patients, and the same percentage of patients with a preoperative LL apex at L5 shifted proximally to L4 at the final follow-up.

No temporary and permanent neurological injuries occurred. Two patients (18.2%) experienced complications. One patient experienced slight PJK, and the rod broke second to pseudarthrosis 7 years after surgery. Another patient suffered from PJK at the 30 months follow up. Both patients underwent revision surgery.

The scores from the SRS-22 survey are summarized in Table 3. No difference of function/activity was found between pre-operation and the final follow-up (*P* = 0.18). However, scores of pain, self-image, and mental health all significantly improved at final follow-up (all *P* < 0.05). Management satisfaction scores were also maintained at follow-up (*P* = 0.887).

**Table 3:** Comparison of SRS-22 questionnaire outcomes preoperatively, postoperatively and at the final follow-up

Parameter	Pre-op	Post-op	Follow-up	*P*
Pain	2.15 ± 0.67	3.88 ± 0.45	4.58 ± 0.47	< 0.001*
Self-image	2.13 ± 0.65	4.45 ± 0.34	4.49 ± 0.58	< 0.001*
Function/Activity	3.75 ± 0.58	3.67 ± 0.38	4.16 ± 0.64	0.18
Mental health	3.40 ± 0.49	4.55 ± 0.40	4.44 ± 0.77	0.005*
Management satisfaction	–	4.45 ± 0.47	4.41 ± 0.77	–

*means significant difference of the parameters between pre-op and final follow-up

## Discussion

SK comprises the majority of sagittal spinal deformity in adolescents. There is a great need for further research on sagittal spinopelvic parameters in SK patients, especially in severe STLK. The medical literature on the post-surgical effects of treatments such as Ponte osteotomy is limited, but suggests few effects on spinopelvic profiles. Currently, there is no research regarding changes in sagittal spinopelvic alignment upon PSO treatment in STLK patients.

It has been suggested that proper sagittal alignment is correlated to health related quality of life in patients with spinal deformities [4, 13, 14]. The analysis of the sagittal spinal alignment is crucial to the proper management of spinal diseases. Our goals during surgical treatment of SK should lay not only on correcting deformity, but also include acquiring a stable and harmonious spinopelvic balance. Scientists claim that different curve patterns have distinct compensatory mechanisms that contribute to the achievement of sagittal balance. Jiang et al [9] speculated that compensation of the lumbar spine was incomplete due to the relatively lower apex in STLK, and therefore the pelvic compensation may play a critical role in the maintenance of sagittal balance. Thus, our study reviewed a cohort of severe STLK patients undergoing posterior-only correction and PSO procedure, and determined how PSO for the correction of severe STLK influences sagittal spinopelvic alignment.

As Lowe et al [15] suggests, kyphosis greater than 65 degrees in the thoracolumbar spine should be treated more aggressively with surgery in SK patients, as they are more likely to progress to aggravating curve, refractory pain, loss of sagittal balance, and neurologic deficit. In agreement with this view, we defined STLK patients with thoracolumbar kyphosis greater than 65° to have severe STLK. Until recently, there have been few studies examining a homogeneous series of patients with severe STLK. Previously published studies document an average 47–54.6° GK in STLK [9, 16]. In the current study, GK was significantly greater than previously found, at 74.1 (69.5, 85.5) pre-operation. This greater magnitude of thoracolumbar area deformity may a have greater influence on sagittal profiles.

In a retrospective study, Ashraf et al [17] reviewed 18 patients with SK and explored changes in sagittal plane alignment following surgery. The authors recorded notable reductions of GK, TK and LL after surgery and no changes in PI, PT and SS. While their study was composed of both typical and atypical SK patients, our study was composed fully of STLK individuals. Similarly, we found significant improvements in terms of GK, TK, TL and LL, and no statistical differences in PI, PT and SS at the final follow-up (Table 2).

As a transition from the thoracic spine to the lumbar spine, the thoracolumbar spine plays an important role in sagittal spinal alignment [18]. We believe it is important to achieve a relatively straight thoracolumbar junction area in severe STLK individuals. In the present study, we found improvement of TL from a median of 49.5° pre-operatively to 8.5° at the final follow-up, which was due to a gentle shifting within thoracolumbar area. Reconstruction of a smooth transition at the thoracolumbar junction was one of the major treatment goals for severe STLK.

Lonner et al [19] reviewed ninety-six surgical SK patients and found the apex localized between T5-T8 in 68.5% of patients with a preoperative apex caudal to T8, whereas 90% of patients with a preoperative apex between T5 and T8 remained unchanged; Changes in thoracic apex location were associated with improvements in the SRS function domain. We noticed a similar changing and restoration of thoracic kyphosis apex and curve shape in our study as well. As shown in Fig. 1, we observed a significant trend wherein the apex of deformity migrates toward the physiological apex, achieved reaching T7 to T8 in most of the cases at the final follow-up. Additionally, TK and LL median 38.4° and 45.1°respectively at the final follow-up, achieving a harmonious correction to optimal physiologic ranges.

A similar phenomenon could be observed in the lumbar curve. Roussouly et al [20] advocated to divide LL into four types. In type 1, the apex of LL is very low, close to L5; LL is short, therefore the kyphosis is long, with an extension on the thoraco-lumbar area; In summary, this causes misalignment with thoracolumbar kyphosis and short hyperlordosis. Type 2 presents with both long and flat LL with a higher position of apex. Type 3 is classified by 35° < SS <45°, apex of LL located at L4, a welly balanced LL, and curve shift between TK and LL located on the thoraco-lumbar area, comprising a normal back. Type 4 has SS >45°, increasing LL with an upper apex and a progressively forward tilt. The majority of the severe STLK patients displayed a low-grade PI, small SS and L5 apex location pre-operation, and could generally be classified as type 1. As Fig. 2 shows, 72.7% of these severe STLK individuals succeeded in achieving movement of the apex of lumbar curve proximally from L5 to L4. SS median 32.7° while TL median 8.5° at the final follow-up, which suggests a smooth thoracolumbar shift from TK to LL, as well as an SS value in accordance with type 3. Overall morphology of the whole spine and pelvis improved to normal parameters.

PI is widely accepted to play an important role in the regulation of pelvic orientation. PI is thought to be anatomical parameter, specific to each individual and independent of any other parameters, while PT and SS are positional parameters and are affected by pelvis rotation direction [21, 22]. In normal individuals, PI is generally reported to be 50° [23, 24]. Li et al [25] reports a significant lower PI value of 35.1° in congenital and chronic tuberculosis populations with angular kyphosis. The authors proposed that angular kyphosis occurring during the growth period could lead to an abnormal pelvic morphology. Although our study consists of etiologically different populations compared to previous studies, we also observed an apparently lower value of PI (median 40.1°) in these severe STLK cases with sharp kyphosis.

SK patients who developed junctional kyphosis appeared to have a significant post-operative lumbopelvic mismatch; LL should be planned according to preoperative PI values to avoid excessive reduction [26]. Lonner et al [27] demonstrated a strong correlation between PI and LL. The authors argued that PI could serve as a reliable factor in predicting expected LL in patients with spine deformities. PI was used to predict the LL needed to obtain sagittal balance for each individual according to the following formula: LL = PI ±9 [24]. Notably, the absolute value of LL- PI significantly improved from a median of 26.5° pre-operatively to 6.1° at the final follow-up in this series, successfully obtaining ideal LL values by adapting to the abnormally lower PI after surgery.

**Fig. 3:**
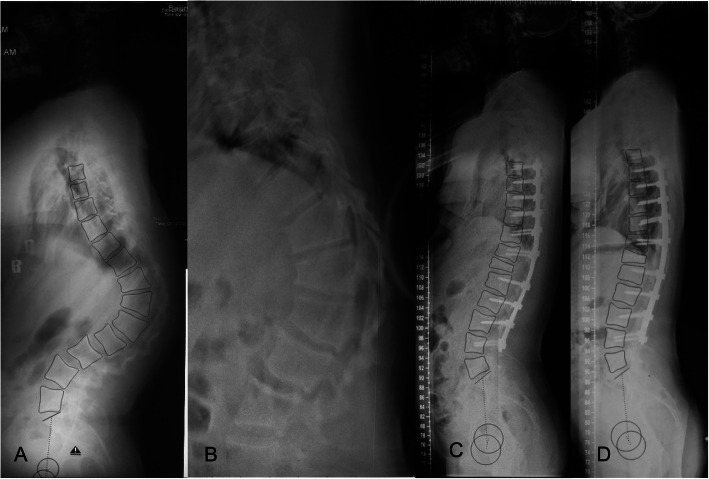
Typical severe Scheuermann Thoracolumbar kyphosis (STLK) case receiving posterior-only correction, PSO and facetectomies with pedicle screw instrumentation

Figure 3 showed a typical case of above sagittal changes. After surgery, Magnitude of TK, TL and LL fell in physiological ranges and apex of thoracic and lumbar migrated to proper physiological locations. Besides, lumbopelvic match could be obviously observed at final follow-up.

As SRS-22 questionnaire scores revealed, great improvement was observed in STLK patients receiving PSO surgery (Table 3). The scores for pain, self-image and mental health dimension improved greatly after surgery and at the time of follow-up, which was consistent with the satisfactory sagittal balance outcomes.

There are two potential limitations in our study. Firstly, this was a retrospective study, which may endanger the strength of the conclusions. Secondly, due to the low prevalence of severe STLK, the sample size is relatively small. Further studies with large-scale sample sizes are still necessary in the future to provide stronger evidence.

Despite these shortcomings, our study has two major strengths. First, it contains a homogeneous group of patients. All patients had severe STLK (greater than 65°) and were operated upon by surgeons from the same institution, with similar operative protocols and surgical technique. Secondly, there are few studies that have similarly focused on studying sagittal spinopelvic alignment after PSO is employed in the treatment of severe STLK.

## Conclusion

In our study of patients with severe STLK receiving posterior-only correction and a PSO procedure with fully pedicle screw construct, we found that although pelvic parameters show no significant changes, there is a reshaped spine alignment toward physiological state (apex of thoracic and lumbar migrated to proper physiological morphology and a delicately thoracolumbar transition from thoracic spine to lumbar spine). Additionally, we found that a harmonious balance was achieved between the spine and pelvis (reaching a relatively low-energy-consumption Roussouly type 3 lumbar curve and proper LL adapted to the abnormally lower PI). However, three column osteotomy is a high risk procedure that should only be done by surgeons sub-specializing in complex spinal deformity correction surgery.

## Data Availability

The datasets generated and analyzed during the current study are not publicly available due risk of compromising individual privacy but are available from the corresponding author on reasonable request.

## Abbreviations

SK: Scheuermann kyphosis
STLK: Scheuermann thoracolumbar kyphosis
PSO: Pedicle subtraction osteotomy
GK: Global kyphosis
TK: Thoracic kyphosis
TL: Thoracolumbar kyphosis
LL: Lumbar lordosis
SVA: Sagittal vertical axis
PI: Pelvic incidence
PT: Pelvic tile
SS: Sacral slope
SRS-22: Scoliosis Research Society-22 questionnaire
